# Design of Monitoring Systems for Contaminant Detection in Water Networks Under Pipe Break-Induced Events

**DOI:** 10.3390/s25175320

**Published:** 2025-08-27

**Authors:** Ludovica Palma, Fatemeh Hatam, Armando Di Nardo, Michèle Prévost

**Affiliations:** 1 Industrial Chair on Drinking Water, Department of Civil, Geological and Mining Engineering, Polytechnique Montréal, CP 6079, Succ. Centre-Ville, Montréal, QC H3C 3A7, Canada; fatemeh-2.hatam@polymtl.ca (F.H.); michele.prevost@polymtl.ca (M.P.); 2 Department of Engineering, Università della Campania Luigi Vanvitelli, 81031 Aversa, Italy; armando.dinardo@unicampania.it

**Keywords:** drinking water, water distribution systems, weighted topological approach, low-pressure events, multi-species analysis, EPANET-MSX

## Abstract

Water distribution networks (WDNs) are critical infrastructure yet vulnerable to contamination, thereby threatening public health. Rapid contaminant detection through sensor systems is essential for water safety. This study compares topological and optimization-based methods for sensor placement under intentional and accidental contamination scenarios triggered by low-pressure events. A novel approach is introduced to model pipe break events that generate low-pressure zones, creating pathways for contamination. Unlike traditional models, this method dynamically estimates contaminant intrusion volume based on the available node pressure. The study reveals that while optimization-based sensor placement yields better outcomes than the topological approach, the performance gap narrows as the number of sensors increases or when the system is tested against scenarios different from those used for optimization. The findings highlight a major issue in sensor detection when water quality is considered. For *E. coli* contamination in a chlorinated system, two conclusions emerge: rapid inactivation of *E. coli* makes it an unreliable indicator, even with optimized sensors, and sensor type and detection thresholds significantly affect performance, requiring careful assessment before implementation. This study provides a framework for evaluating sensor systems in WDNs, emphasizing tailored strategies that consider hydraulic conditions and water quality dynamics to improve contamination detection and public safety.

## 1. Introduction

Water distribution networks (WDNs) are vital infrastructure systems, critical for delivering safe and clean water to communities, thus playing a key role in maintaining public health. Ensuring water quality within these networks is a complex task due to multiple contamination risks. Contaminations may be accidental—caused by low-pressure events that allow external contaminants to enter through breaks or leaks [1]—or intentional, such as in the case of terrorist attacks [2]. Real-time sensors can enable rapid contaminant detection, but identifying their optimal placement remains a complex challenge.

Numerous studies have proposed methodologies employing optimization techniques to address this issue; a detailed literature review can be found in Adedoja et al. [3]. While potentially effective, these methods are often computationally demanding, making them impractical for large-scale WDNs, especially considering the lack of calibrated hydraulic and water quality models available to many water utilities.

In contrast, topological methods avoid hydraulic simulations, focusing on structural features like centrality [4,5]. Santonastaso et al. [6] compared empirical, topological, and optimization-based strategies, finding optimization to be slightly superior but highlighting the efficiency of topological methods. However, their analysis focused solely on intentional arsenic contaminations, without testing sensor performance across diverse contamination scenarios. This limitation is particularly relevant for the optimization approach, where sensor placement is tailored to the simulated arsenic contamination events and may not generalize well to other types of intrusion events, such as contaminations caused by low-pressure events.

To compare the different approaches proposed in the literature, it is essential to highlight a key feature of the optimization approaches: they rely on simulated contamination scenarios to determine sensor locations. This involves modeling different types of contaminants and using the outcomes to identify optimal sensor placements. However, these simulations carry an inherent uncertainty, as real-world contamination events may deviate from the modeled scenarios. Therefore, the reliability of sensor placements derived from optimization models in actual contamination incidents remains questionable. In contrast, topological approaches do not require contamination simulations. They represent the WDN as a graph and use structural network properties to define sensor placement.

As discussed by Palma et al. [7], regardless of the approach used, most studies in the literature on sensor placement assume the use of generic sensors capable of detecting all types of contaminants [8]. However, this assumption does not reflect the reality of commercially available sensors, typically designed to monitor only specific water quality or hydraulic parameters. Berglund et al. [9] highlighted that among over 200 studies on emergency management, only 12 used multi-species analysis to model realistic water quality dynamics. Yet, to detect contaminants, especially those that react with disinfectants, accounting for water quality processes is essential. Effective detection requires a thorough evaluation of sensor feasibility, considering both contaminant–disinfectant interactions and the specific capabilities of available sensors.

Modeling both intentional and accidental contamination presents unique challenges. Intentional intrusions are more commonly studied [10,11], but accidental ones, linked to low pressure or pipe failures, are underexplored. Taylor et al. [12] linked pressure, leakage, and intrusion volume in intermittent water supply systems, modeling pressure at leakage points as an unknown probability distribution. Odhiambo et al. [13] modeled contaminant intrusion from pipe breaks but assumed intrusion volume based on an additional demand rather than actual pressure data. As highlighted by Rathi [14], there remains a notable gap in the literature regarding the simulation of accidental contamination intrusions caused by leakage events. These contaminations would first require modeling of a low-pressure event to create a pressure gradient, followed by the simulation of contaminant ingress.

When simulating accidental contaminations, the contaminant load entering a WDN depends on both the external concentration and the volume of water intruding into the system. Many previous studies have relied on simplified assumptions that decouple these variables from real-time network dynamics. For instance, Betanzo et al. [15], Teunis et al. [16], and Islam et al. [17] modeled intrusion events using fixed or randomly assigned contaminant mass flow rates, volumes, or resulting contaminant concentrations within the pipe independent of the actual hydraulic conditions. Besner et al. [18] used probabilistic models based on pressure differentials to estimate a range of potential mass flow rates. While these models provide a broad understanding of contamination impacts, they often fail to account for spatial and temporal variations in nodal pressure that govern actual intrusion behavior. Hatam et al. [19] addressed the difficulty of estimating these variables by proposing an event-specific method that calculates intrusion flow at each node based on local pressure. While accurate, this approach is computationally intensive.

Pipe break simulations in WDNs have been explored in the literature. Cornell University developed GIRAFFE, a tool integrated with EPANET to simulate pipe breaks [20]. Klise et al. [21] used WNTR to model earthquake impacts on WDNs, incorporating damage to pipes, tanks, and pumps to assess system resilience. However, despite the frequent occurrence of pipe breaks in water networks and the associated risks of accidental contamination, there is a notable lack of studies that explicitly simulate contaminant ingress from such events. Moreover, contaminant detection studies often rely on generic sensors, overlooking the limitations of commercially available devices and ignoring interactions between contaminants and disinfectants, leading to inflated contamination estimates and unrealistic risk assessments. Furthermore, research comparing optimization and topological approaches for sensor placement rarely assesses performance across different contamination types.

This study addresses existing gaps by comparing topological and optimization-based methods for sensor placement in a real-world WDN. Sensor placements are evaluated through simulations of different contamination scenarios, with a novel approach to model pipe breaks and intrusion without predefined contaminant volumes. This study integrates hydraulic and water quality analysis using EPANET-MSX and simulates the effectiveness of pressure, *E. coli*, and chlorine sensors in detecting accidental contamination. By addressing these key gaps, this study not only introduces a novel methodology for simulating accidental contamination but also offers a robust framework for evaluating sensor placement strategies in water distribution networks. These contributions enhance understanding of both hydraulic disruptions and water quality impacts, improving risk management and contamination detection capabilities.

## 2. Materials and Methods

### 2.1. Sensor Placement Strategies

This study compares optimization-based and topological methods for determining optimal sensor locations. In the topological approach, the water distribution network was modeled as a graph G=(V,E), where V represented nodes and E represented the connecting pipes. Various weighting schemes were applied, based on pipe length, diameter, elevation, and hydraulic conductance, to assess their impact on sensor placement. Length-based weighting explored both long pipes that acted as main conduits and short, central pipes that supplied areas with a higher number of nodes. High-conductance pipes were prioritized under the assumption that faster flow may facilitate earlier detection, while elevation-based weighting targeted high-altitude nodes, which are more prone to pressure drops during low-pressure events. The objective was to determine whether specific weighting factors consistently enhanced detection or if performance varied depending on the contamination type and sensor count. All weighting schemes, along with the unweighted case (Table 1), were tested to leverage the computational efficiency of the topological approaches.

Once the WDN was represented as a graph, spectral clustering was applied [23], assuming the number of clusters was equal to the number of sensors to be placed. For each cluster, the sensor location was then defined according to the edge betweenness centrality. The centrality of a node u is determined by how often it lies on the shortest paths between other nodes in the network. It is calculated by evaluating the total number of shortest paths that pass through u relative to all possible shortest paths in the network. A node with higher centrality plays a more critical role in network connectivity, as it frequently serves as an intermediary in the most efficient routes between different points. It is important to note that since it uses the shortest path concept, the weights defined before needed to be reversed for calculation purposes. In this way, the shortest path became the one characterized by the highest weight. Finally, for each cluster, the sensor was placed at the most central node. To test the performances of the developed sensors system with respect to the simulated contaminations, the probability of detection (DP) was estimated. *DP* is defined as the ratio of detected scenarios to the total number of simulated scenarios ($DP=N_{ds}/N_{s}$).

The optimization approach in this study was single-objective, aiming to maximize the *DP*. A Genetic Algorithm (GA) was used to determine the optimal sensor placements according to this objective. The GA was configured with a population size of 200 individuals and 200 generations, default crossover and mutation probabilities, defined through a trial-and-error approach. The topological analysis was performed on a laptop with an 11th Gen Intel (R) Core (TM) i7-11850H processor @ 2.50 GHz and 64 GB of RAM. The optimization phase was conducted through Calcul Quebec, utilizing two nodes equipped with 40 cores and 3 GB of memory each, featuring an Intel Gold 6148 Skylake CPU @ 2.4 GHz and 6 × 480 GB SSDs.

### 2.2. Contamination Scenarios

The effectiveness of the developed sensor systems was evaluated through simulations of various contamination scenarios, as described below. All the simulation scenarios were generated using the EPANET-Matlab Toolkit (Version 2.2.6.1) [24].

Scenario set IC—Intentional contaminations. Intentional contamination scenarios considered the introduction of a conservative tracer at each network node to simulate potential threats to water distribution systems. In each scenario, the contaminant was represented as a mass source and injected at a flow rate of 125 L/h with a concentration of 230,000 mg/L over a one-hour period [25].

**Figure 1:**
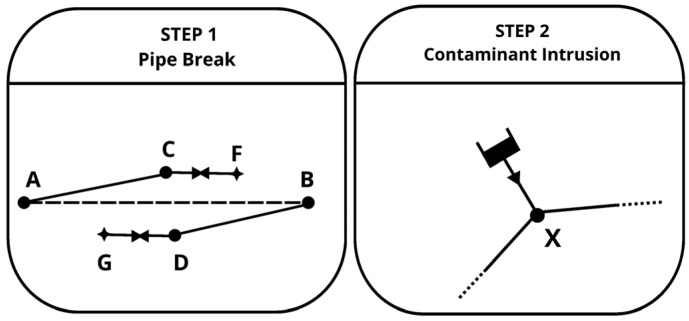
Creation of accidental intrusion scenarios.

Scenario set AC-NC—Accidental contaminations in non-chlorinated WDN under low-pressure conditions. A necessary condition for accidental contamination is the existence of a negative pressure gradient, where the external pressure exceeds the internal pressure in the pipeline. In this study, accidental contamination scenarios resulting from pipe breaks were simulated in two stages: first, the pipe break event, followed by the intrusion of contaminant into the network (Figure 1).

Pipe Break:

A pipe break was simulated as a full disconnection at the pipe’s midpoint, allowing water to escape from both ends. The simulation of a pipe break was carried out through the following steps:

1. The original pipe AB was closed for the duration of the pipe break.
2. Two new nodes, C and D, were added with the same elevation, equal to the average elevation of nodes A and B.
3. Two new pipes, AC and BD, were introduced, both having the same diameter and roughness as the original pipe AB. The length of the two new pipes was set as $\bar{AC}=\bar{BD}=\bar{AB}/2.$ The roughness coefficient of the fictitious pipe was set to 100,000, and the minor loss coefficient was set to 1, ensuring that all energy loss from the leak was attributed solely to the minor loss [20].
4. Two emitter nodes, F and G, were added with elevations equal to those of nodes C and D plus 1 m, representing the water level outside the pipe. The emitter values were determined using the orifice equation.
5. Flow control valves (FCVs) were installed on the fictitious pipes connecting node C to emitter F and node D to emitter G. FCVs were added to prevent unrealistic flow rates during pipe breaks.

To generate a comprehensive dataset, breaks were assumed to occur in any pipe within the network. These simulations identified low-pressure events, defined as nodes experiencing pressure below 1 m, which were considered as intrusion points. In these simulations, single-node intrusions were assumed at a time, meaning that when multiple nodes experienced pressure below 1 m, the model was run separately for each, generating an individual intrusion scenario for each affected node. The pipe break was assumed to last 4 h during peak demand.

Intrusion Simulation:

To estimate the resulting contaminant concentration within the network following an intrusion event, two key factors must be considered: (i) the concentration of the contaminant outside the pipe (e.g., in surrounding soil or water) and (ii) the volume of contaminated water entering the pipe (which depends on the local pressure deficit).

In prior studies, contaminant concentrations within the network were often predefined, without explicitly modeling intrusion dynamics. In contrast, the approach introduced in this study used a fictitious reservoir to represent the external contamination source. This allowed for a dynamic, pressure–responsive estimation of intrusion flow during pipe break events. By directly linking intrusion flow to the real-time hydraulic state of the network, and combining it with known external contaminant concentrations, the model allowed for a more realistic calculation of the resulting in-pipe concentration. This represents a methodological advancement, as it explicitly models the interplay between pressure-driven intrusion and contaminant transport in a fully coupled manner—without relying on fixed assumptions or iterative approximations.

The reservoir was positioned 1 m above the intrusion node elevation (simulating the water level outside the pipe) and it was connected via a very short pipe (0.1 m) with a Hazen–Williams roughness coefficient set to a high value (100,000), a small internal diameter of 1 mm, a minor loss coefficient equal to 1; a check valve was included to ensure unidirectional flow into the network. These parameters were carefully chosen to ensure that all energy loss from the leak was attributed to a minor loss, thereby reproducing the localized hydraulic behavior of a small leak or orifice [20].

In the proposed methodology, the intrusion flow is directly computed by EPANET’s hydraulic solver based on the head difference between a fictitious reservoir and the intrusion node. EPANET computes head loss across the pipe using the Hazen–Williams formulation, augmented by a term for minor losses:

$$h_{L}=10.67\cdot \frac{L}{C^{1.852}\cdot D^{4.871}}\cdot Q^{1.852}+K\cdot \frac{v^{2}}{2g}$$

In this equation, $h_{L}$ is the total head loss between the reservoir and the node, *L* is the pipe length, *C* is the Hazen–Williams roughness coefficient, *D* is the pipe diameter, *Q* is the flow rate, *K* is the minor loss coefficient, *v* is the flow velocity, and *g* is the gravitational acceleration [26]. Under normal conditions, both friction and minor losses contribute to the total head loss. However, in this configuration, the contribution of the first term becomes negligible. This is because the pipe is exceptionally short (reducing the impact of length *L*), the roughness coefficient is very high (which increases the denominator), and, critically, the diameter is small (1 mm), which raises the denominator to a power of 4.871. These combined effects render the entire friction term close to zero, regardless of flow rate.

As a result, head loss is dominated by the minor loss term, which depends solely on the local velocity at the entrance. Since the flow velocity *v* is related to the flow rate through the cross-sectional area of the pipe, the resulting relationship between the flow *Q* and the pressure head difference Δ*h* across the pipe becomes proportional to the square root of Δ*h*. This mirrors the form of the classical orifice equation, and although EPANET does not explicitly solve this orifice equation, the combination of physical parameters used in the pipe setup ensures that the system behaves equivalently. EPANET’s solver internally adjusts the flow at each time step based on the hydraulic conditions, allowing for the intrusion to respond dynamically to pressure variations in the network [26]. This approach eliminates the need for external iterative procedures or predefined intrusion profiles, and provides a scalable, physically grounded way to simulate contamination ingress under low-pressure conditions in large water distribution networks.

A conceptual framework diagram illustrating the entire procedure (from pipe break simulation to single-point intrusion modeling) is provided in the Supplementary Materials (Figure S1).

The concentration of *E. coli* outside the pipe was assumed to be 1% of the maximum *E.coli* concentration in raw wastewater in Montreal Urban Community [27], and so, was set to 6200 CFU/100 mL.

Scenario set AC-C—Accidental contaminations in chlorinated WDN under low-pressure conditions.

Contamination categories IC and AC-NC offered insights into general contaminant spread but excluded interactions with water quality parameters. Scenarios AC-C simulated accidental contamination from pipe breaks under low-pressure conditions, focusing on the 100 worst-case events with the highest contaminant mass. These scenario sets incorporated chlorine’s role in the WDN, acting as a barrier that limited contaminant spread. To capture disinfectant–contaminant dynamics, multi-species modeling using EPANET-MSX was employed, providing a more realistic assessment of sensor performance in chlorinated networks. Chlorine decay was modeled as in Hatam, Besner, Ebacher and Prévost [19]. Additionally, unlike generic tracer detection, these scenarios accounted for the detection limits of *E. coli*, chlorine, and pressure sensors based on commercially available technologies and evaluated how detection performance varied with different threshold settings.

## 3. Case Study

**Figure 2:**
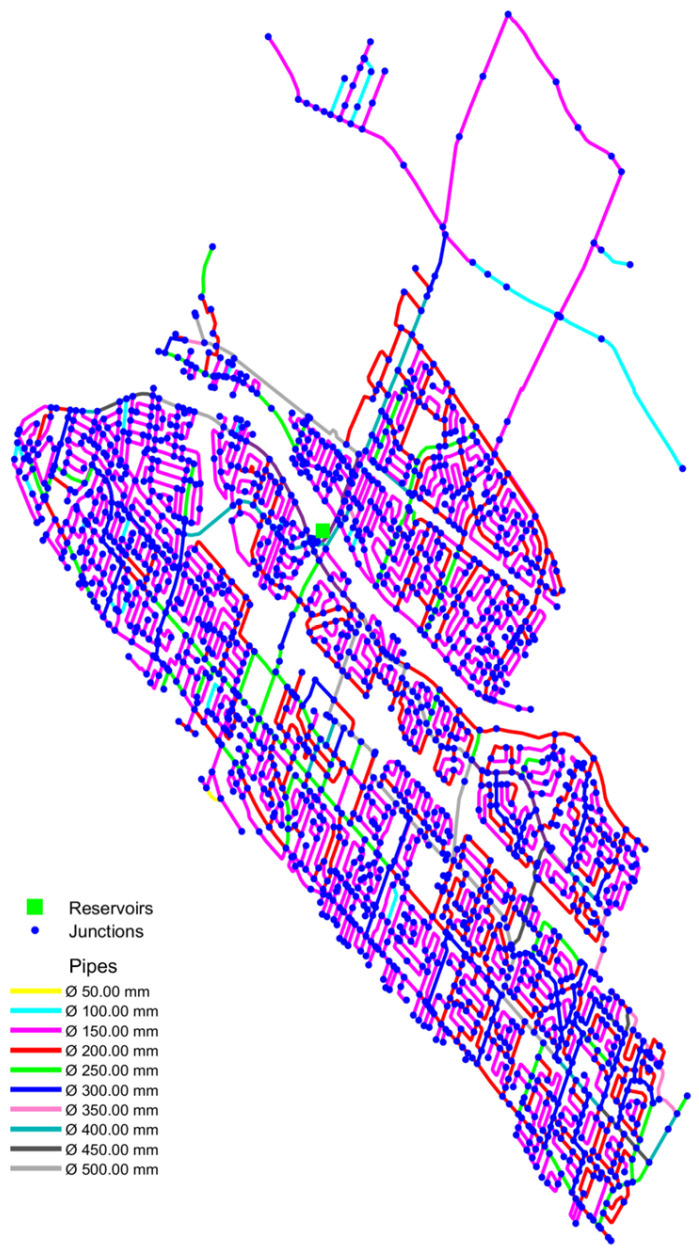
Graphical representation of the WDN with pipes color-coded by diameter.

The testing phase used a real Canadian WDN with 1889 demand nodes, 2640 pipes, and one treatment plant modeled as a reservoir (Figure 2). Simulations were performed in EPANET 2.2 over 7 days, using 5 min time steps and a pressure-driven analysis (required pressure: 14 m; exponent: 0.5). Contaminations were simulated at hour 114 (peak demand) to ensure hydraulic stability and sufficient sensor response time. Three sensor configurations (10, 25, and 100 sensors) were tested to assess detection probability. In the analyzed WDN, an accurate hydraulic model and detailed data, such as historical pipe breaks and reservoir capacity, enabled both intentional and accidental contamination simulations. Historical data on pipe breaks from 2020 to 2023 provided by the water utility included records of break dates, pipe diameters, and break types. For medium-to-small pipes (<400 mm), circumferential breaks were common, whereas large-diameter pipes (>400 mm) did not experience circumferential breaks, aligning with the findings of Gharaati and Dziedzic [28]. Based on this, pipe breaks in small-to-medium pipes were simulated as circumferential failures, while for large-diameter pipes, a maximum equivalent hole diameter of 400 mm was assumed in the simulations.

Since EPANET models reservoirs as unlimited sources, FCVs were introduced to cap flows and reflect real conditions. The treatment plant’s normal outflow was 33,240 LPM. In a pipe break, an additional 30,000 LPM was assumed, regulated by a local FCV. For a 4 h break, this yielded up to 15,176 m^3^—nearly draining the 19,000 m^3^ reservoir.

## 4. Results and Discussion

### 4.1. Tracer Intrusions and Use of Generic Sensors

This section presents the sensor system’s performance in detecting tracer intrusions under both intentional and accidental contamination scenarios (IC and AC-NC). The IC scenario set includes 1890 simulated intrusions, while accidental contamination comprises 1582 events. These are derived from 63 pipe breaks that caused at least one node to experience low pressure (<1 m). Each low-pressure node is treated as a unique intrusion point, resulting in distinct scenarios. Even when the same node is affected across different break events, each case is considered separately due to differing hydraulic conditions and intrusion volumes. Table 2 summarizes the DP of the different sensor systems for both topological and optimization approaches across scenario sets IC and AC-NC, with corresponding visualizations provided in Figure S2. In this part, a contamination event is classified as detected if at least one sensor registers a non-zero tracer concentration at any point during the simulation, regardless of the detection time, under the assumption of generic tracer sensors. For each scenario set, DP is assessed for sensor deployments of 10, 25, and 100 sensors. Given that the WDN consists of 1889 demand nodes, these configurations correspond to sensor coverage rates of approximately 0.5%, 1.3%, and 5.3% of the network. Moreover, for the topological approaches, the coefficient of variation (CV), defined as the ratio of the standard deviation to the mean, is calculated to assess the sensitivity of sensor placement to different weighting factors, highlighting how results fluctuate based on the chosen weight.

Sensor placement optimization is first performed using scenario set IC, and the resulting sensor configuration is evaluated by comparing *DP* on both IC and AC-NC. This process is then repeated by optimizing on AC-NC and evaluating *DP* on both sets.

Assessing the influence of weights and number of sensors in topological approaches. The topological approaches show CV values of 6%, 9%, and 3% for IC scenarios with 10, 25, and 100 sensors, respectively, while for AC-NC scenarios, they are 8%, 10%, and 3%. Although the CV remains relatively low overall, the choice of weighting factor still influences the *DP* when fewer sensors (10 or 25) are used. However, as the number of sensors increases to 100, the CV drops to 3%, showing that the impact of weighting factors becomes negligible at higher sensor densities.

This trend can be attributed to the fact that a higher number of sensors ensures more uniform spatial coverage, thereby reducing variability in detection performance across different topological weighting factors, as shown in Figure S2. With a lower number of sensors (10 or 25), each sensor’s placement is influenced by the weighting scheme (e.g., prioritizing diameter vs. length), which can lead to substantial variation in DP. However, when 100 sensors are deployed, they effectively saturate the network’s key locations, covering all major topological features regardless of the weighting used. At that point, the influence of individual factors like pipe length or diameter diminishes, and network connectivity becomes the dominant driver of detection performance. This convergence leads to reduced variability in *DP* and a lower CV across weighting strategies, as a higher number of sensors ensures broader coverage of monitoring areas regardless of the initial weighting scheme.

When 10 sensors are placed, for AC-NC, the highest DP (53.79%) is achieved using the inverse of length, yet using length itself produces a nearly identical result (52.53%), both outperforming the no-weight approach (44.88%). This indicates that both long and short pipes contribute strategically to early contamination detection. Long pipes function as primary conduits, facilitating widespread contamination monitoring, while shorter, centrally located pipes often connect high-demand nodes, making them critical detection points before contamination propagates further.

For IC, the best-performing weight is the inverse of diameter for 10 sensors. In the WDN model, the smallest available pipes have a diameter of 150 mm. By assigning a weight inversely proportional to the diameter, this approach highlights both the connectivity of the pipes (inherent to the topological method) and the smaller pipes. Consequently, the sensor system prioritizes these small, highly connected pipes, which act as critical junctions in the network, linking large portions of the system. Placing sensors exclusively on large diameter pipes would fail to detect contaminations occurring downstream, as these generally have more downstream than upstream nodes, leading to potential blind spots. In contrast, sensors positioned on smaller diameter, but well-connected pipes, balance upstream and downstream contamination risks, improving overall detection performance. In the IC with 25 sensors, the highest *DP* is achieved with the unweighted approach. However, when using the inverse of diameter, inverse of length, or elevation as weights, the results are almost identical. This indicates that the placement of sensors on large, main pipes (with high diameter, hydraulic conductance, and length) significantly lowers the DP. When sensors are placed in smaller, well-connected pipes, the weight choice becomes less important. In this case, network connectivity becomes the dominant factor influencing sensor performance.

Table 2 highlights the key trade-offs between sensor deployment and detection performance. Increasing the number of sensors from 10 to 25 resulted in a significant improvement in *DP*, particularly for the topological approaches. While increasing the number of sensors from 25 to 100 improves *DP* by a maximum of approximately 24%, the benefit must be balanced against the significant costs associated with purchasing, maintaining, and managing data from a larger sensor network.

Using elevation as a weighting factor did not significantly enhance the detection of accidental contaminations. This is likely due to the network’s topology, where the highest elevation nodes, are predominantly located at the network’s periphery rather than in central areas. As a result, these nodes are not critical junctions in the network’s overall connectivity, limiting their influence in a topological-based sensor placement strategy. Consequently, the results obtained with elevation-based weighting closely resemble those of the unweighted approach.

No single weighting factor consistently shows better performances than the others across all conditions. The optimal choice varies depending on the contamination scenario and the number of sensors deployed. Given the computational efficiency of the topological approach, water utilities could feasibly test multiple weighting strategies and select the most suitable one based on sensor availability and the most critical contamination risks. Alternatively, they may opt for a robust weighting factor that, while not necessarily optimal in every scenario, consistently delivers strong performance, such as the inverse of length, which provided optimal or near-optimal solutions across multiple cases.

Future research is recommended to investigate whether the influence of different weighting factors remains consistent across various types of WDNs. Additionally, studies with an increased number of sensors could help determine if there is a threshold beyond which weighting factors lose significance and connectivity becomes the dominant factor in sensor placement.

Topological vs. Optimization. When comparing these two approaches, the optimization method consistently achieved higher DP than the topological approach. However, the practical benefit of this superiority diminishes as the number of sensors increases. For example, with 25 sensors, the best-performing topological approach (inverse of diameter) achieved a DP of 74.97% with respect to accidental scenarios, while the optimization approach tailored for accidental contaminations reached 80.34%. However, when applying an optimization approach designed for scenarios IC to scenarios AC-NC, the DP is 77.88%, which is closer to the topological approach. In this latter case, while the optimization method still performs better, the improvement is marginal (2.9%), raising the question of whether the significantly higher computational effort and complexity of optimization-based placement are always justified. Moreover, while the optimization approach consistently achieves a DP above ~70% regardless of the number of sensors, the performance of the topological approach is highly sensitive to sensor density.

**Figure 3:**
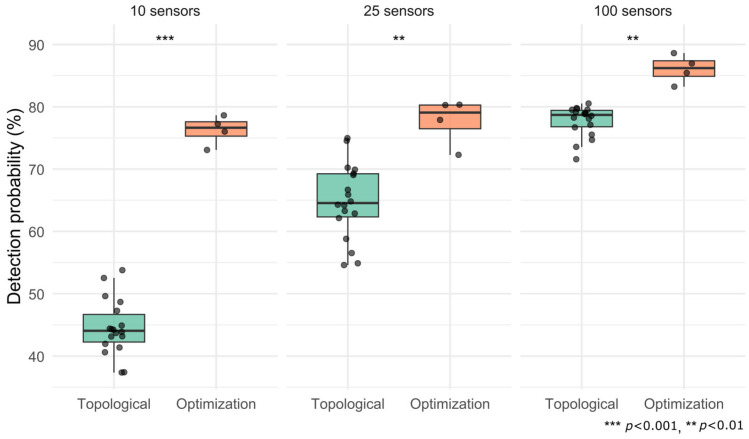
Detection probability for topological (green) and optimization (orange) methods under combined IC and AC-NC scenarios, evaluated for systems with 10, 25, 100 sensors. Wilcoxon’s test: *** *p* < 0.001; ** *p* < 0.01.

To evaluate whether the higher DP achieved with optimization-based placement is statistically significant, the DP distributions of the two methods (topological vs. optimization) are compared for each sensor density using a two-sided Wilcoxon rank-sum test performed in RStudio (version 2024.09.1). The statistical analysis was conducted by grouping results from the IC and AC-NC scenarios together, and then dividing them based on the sensor placement method and the number of sensors (10, 25, and 100). Figure 3 shows the corresponding box-and-whisker plots with jittered points for each variant and the significance labels (***, **). For 10-sensor systems, the DP gap is highly significant (*p* < 0.001), reflecting a significant median improvement. Even with 25 and 100 sensors, the gap remains significant (*p* < 0.01), although the distributions begin to overlap, indicating that increased sensor density mitigates, but does not eliminate, the advantage of optimization.

The results demonstrate that optimization-based sensor placements can be highly scenario-specific. Their advantage over topological approaches diminishes when tested on scenarios different from those they were optimized for. Additionally, Figure 3 suggests that for sufficiently dense sensor deployments, even computationally simpler methods like the topological approach can achieve results comparable to those of complex optimization techniques. Moreover, the topological approach is computationally efficient, requiring only 2–4 min for the studied network to determine sensor placements. However, the optimization approach is widely recognized as computationally challenging, particularly for large-scale networks [29,30]. Indeed, in this study, the optimal sensor placement defined with GA, even with powerful computing resources, required a computational time between 6 and 23 h to produce results. This comparison underscores the computational trade-off between the two approaches: the topological method achieves rapid results within minutes on standard hardware, whereas the GA-based optimization requires significantly longer runtimes, even on advanced high-performance computing infrastructure, due to the complexity of evaluating and evolving sensor configurations. This study adopts a single-objective optimization strategy to allow for a direct comparison with the topological approach. While the GA parameters were defined based on empirical tuning, a more systematic sensitivity analysis, as well as the exploration of alternative or multi-objective optimization frameworks, could further enhance robustness, and are recommended for future work.

The comparison between topological and optimization methods aligns with the findings of Santonastaso, Di Nardo, Creaco, Musmarra and Greco [6]. While their study focused solely on arsenic contamination, assuming uniform contamination across all nodes, this work expands the analysis to multiple contamination types. Optimization-based placement demonstrated higher detection performance compared to the topological methods. This suggests that in real-world situations, where actual contamination events may differ from simulated ones, the topological method offers an adaptable and reliable solution. Additionally, the topological approach does not require a fully calibrated hydraulic and water quality model, which the optimization method relies on. Any errors in the model can significantly impact the optimization results. Optimization approaches can be highly effective when contamination scenarios are well-defined and accurately modeled. This requires a well-calibrated, continuously updated WDN model and detailed information on potential events. However, these methods are computationally intensive, particularly for large networks, where generating all possible scenarios and determining optimal sensor placements can result in significant time and resource demands. Optimization can deliver not only highly precise but also computationally feasible solutions for WDNs with manageable complexity and where the computational effort is justified by the certainty of the scenarios modeled. For larger, more complex systems or where uncertainty is high, simpler, more adaptable methods like the topological approach may offer a more practical solution, especially when balancing accuracy with feasibility.

Therefore, for water utilities, this raises important considerations about the trade-off between advanced, resource-intensive modeling approaches that may produce more accurate results (if the models and simulations are reliable) and simpler, faster methods like the topological approach that are easier to implement and still provide solid results, especially with an adequate number of sensors.

### 4.2. Reactive Contaminant Intrusions and Use of Hydraulic and Water Quality Sensors

**Figure 4:**
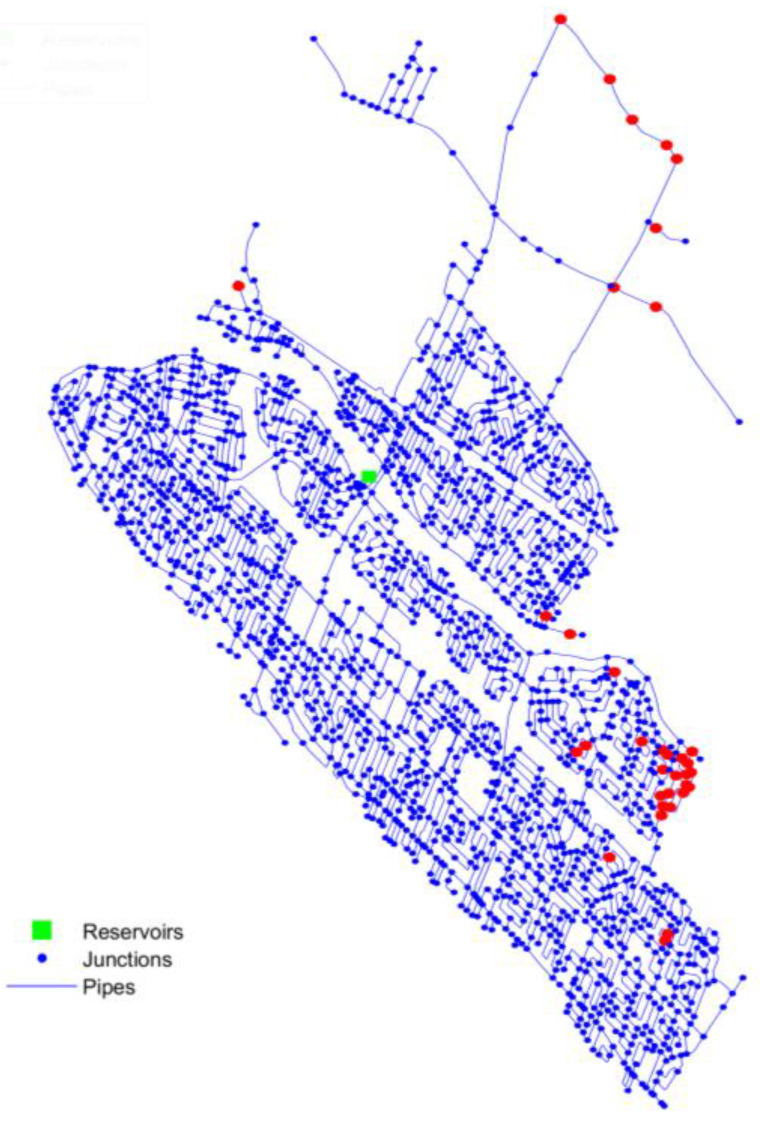
Spatial representation of the WDN highlighting intrusion nodes associated with the 100 worst-case contamination scenarios (red nodes).

In this section, the contamination scenarios from the AC-C set are considered, with the contaminant no longer treated as a passive tracer. The model assumes a steady source concentration of 1 mg/L free chlorine in the WDN, with *E. coli* inactivation by chlorine included in the simulations. Figure 4 illustrates the 100 worst-case events for the AC-C scenario set, with intrusion nodes marked in red, representing the locations with the highest contaminant mass entering the WDN. For these intrusion events, a combination of hydraulic and water quality sensors is considered to assess the performance of specific sensor systems. For pressure, sensors are set to trigger an alarm when levels drop below 3 m. The chlorine variation (ΔCl_2_), defined as the difference between the chlorine concentration under normal conditions and during intrusion scenarios, is used to set the alarm threshold, with two values analyzed: 0.2 mg/L and 0.3 mg/L. In the model, real-time sensors for *E. coli* with two different detection limits (0.1 CFU/L and 0 CFU/L) are also investigated.

Although there is no universally defined ΔCl_2_ threshold that conclusively indicates a contamination event, this study adopts a value of 0.2 mg/L as a practical starting point for intrusion detection by chlorine sensors. Smaller variations often fall within the range of daily fluctuations observed in chlorine residuals across water distribution systems. To account for this, a higher threshold of 0.3 mg/L is also evaluated to assess the sensitivity to more pronounced deviations, as a 0.2 mg/L drop may still occur under normal operating conditions and could trigger false positive alarms. Determining the minimum chlorine variation that indicates an abnormal condition—or a potential intrusion—is inherently challenging, as it depends on factors specific to each distribution system, such as demand patterns, hydraulic and water quality dynamics, and operational practices. To accurately define this threshold, it is essential for water utilities to conduct dedicated data collection and analysis to distinguish between normal fluctuations in residual chlorine and variations indicative of contamination events. For *E. coli* sensors, the detection limits of 0 and 0.1 CFU/L are chosen to explore the impact of slight changes in detection sensitivity on detection probability, starting with the lowest possible threshold and assuming a conservative testing scenario. These threshold values are not intended as regulatory cutoffs or minimal disinfectant residual to be maintained, but rather as representative points to evaluate the performance and responsiveness of different sensor configurations. Moreover, the goal is to illustrate how even small changes in this parameter can meaningfully influence detection probability, emphasizing the need for careful threshold selection during sensor deployment and analysis.

To estimate the efficiency of the sensor systems, *DP* is calculated for each parameter, and it is assumed that all sensors operate continuously, with a detection time step of 5 min.

The 100 worst-case scenarios, as illustrated in Figure 4, are predominantly concentrated in two specific regions of the network. These areas naturally experience lower pressure even under normal operating conditions, making them more vulnerable to further drops after a pipe break and increasing the pressure differential that drives contaminant intrusion. Additionally, these regions are situated at the periphery of the WND and exhibit a low level of looped connectivity. Depending on the local network configuration, a pipe break in these areas may reduce the availability of alternative flow paths, potentially exacerbating pressure drops. The performance of the sensors systems, in terms of *DP*, are summarized in Table 3 and Figure S3. While several topological weighting strategies were evaluated, only two representative methods—relative length and its inverse—are presented in the main results. These are selected based on their consistent performance and to streamline interpretation. A complete summary of detection probabilities for all tested weightings is available in the Supplementary Material (Table S1), which confirms the low variability across methods (standard deviation of 3.9% for pressure sensors and 0–1% for water quality sensors).

In this analysis, five distinct optimization strategies are implemented: (1) optimized for pressure-based DP—sensor locations are optimized to maximize the probability of detecting pressure drops caused by intrusion events (2) optimized for chlorine-based DP (0.2 mg/L)—sensor locations are selected to maximize the probability of detecting chlorine concentration changes above a 0.2 mg/L threshold (3) optimized for chlorine-based DP (0.3 mg/L)—optimization focuses on maximizing the probability of detecting chlorine changes above a 0.3 mg/L threshold. (4) Optimized for *E. coli*-based DP (0.1 CFU/L)—sensor locations are selected to maximize the probability of detecting *E. coli* concentration above a 0.1 CFU/L threshold (5) Optimized for *E. coli*-based DP (0 CFU/L)—sensors are placed with the objective of maximizing the probability of detecting *E. coli* concentration above a 0 CFU/L threshold. Each strategy is used to optimize sensor placement for a specific parameter and threshold, with the resulting configurations evaluated across all parameters to assess their overall detection effectiveness in relation to different sensor types.

A key observation from Table 3 is the stark contrast in DP for water quality sensors compared to the previous scenarios in Table 2, which modeled accidental contamination using a conservative tracer without chlorine and with generic sensors. Specifically, in Table 3, the *E. coli* sensors, now reflecting the interaction with chlorine, show extremely low DP, with a maximum of only 4% for detection thresholds higher than 0 CFU/L. In contrast, Table 2 shows that with generic sensors detecting a tracer at non-zero concentrations, the same topological approach (Inverse of RL) achieved a DP of 43.12%, over ten times higher than when accounting for the interaction between chlorine and *E. coli* in Table 3. Chlorine sensors achieve a maximum *DP* of 3% at a detection threshold above 0.2 mg/L, while optimization-based sensor placement improves these values, reaching a maximum *DP* of 57% for *E. coli* and 11% for chlorine. A more stringent detection threshold (0 vs. 0.1 CFU/L) leads to a decline in *DP*, from 57% to 38%. Notably, when the chlorine-based threshold increases from 0.2 mg/L to 0.3 mg/L, the *DP* drops to just 2%. This highlights the critical importance of defining accurate sensor thresholds during both the development and testing phases, as well as in real-time data analysis, to minimize false positives and false negatives [31].

When the optimization is performed to place 10 chlorine sensors configured to detect variations greater than 0.3 mg/L, only 4 out of the 100 worst-case intrusion scenarios are detected. This suggests that, for the tested scenarios defined by single pipe break events, chlorine variations remain minimal, with only 4 nodes experiencing a chlorine variation higher than 0.3 mg/L. Therefore, for the specific WDN and contaminant intrusions, continuous disinfection effectively compensates for the disturbance in chlorine levels determined by *E. coli* intrusion following pipe break events. Moreover, this performance is achieved on a limited set of worst-case pipe break scenarios, raising concerns about its effectiveness in broader or less intense events. The low detection probabilities of *E. coli* and chlorine sensors observed during contamination events can largely be attributed to the rapid and effective inactivation of *E. coli* by chlorine within studied scenarios. In the analyzed chlorinated network, *E. coli* is quickly inactivated, which limits its effectiveness as an indicator of intrusion. Additionally, in single-node, single-break scenarios, chlorine levels showed minimal variation, as even small residuals were sufficient to inactivate *E. coli*. This is consistent with the findings of Hatam, Besner, Ebacher and Prévost [19], who demonstrate that even under more extreme conditions, the likelihood of detecting *E. coli* in a chlorinated system is extremely low.

In contrast, hydraulic sensors exhibit strong performance in detecting low-pressure events associated with pipe breaks, consistently achieving high *DP* values across both topological and optimization-based approaches. This underscores the reliability of pressure sensors in identifying events indicative of potential contaminant intrusion [32,33].

The limited effectiveness of the topological approach in this case is expected, given that the 100 worst-case intrusion scenarios are concentrated in a specific region of the network. The rapid inactivation of *E. coli* by chlorine prevents significant contaminant propagation, meaning that contamination remains highly localized. Since the topological approach distributes sensors based on network structure rather than contamination risk, it lacks the spatial targeting necessary to detect events concentrated in a single area. As a result, only a small fraction of these high-risk scenarios is captured. In the studied case, the 100 worst-case scenarios are estimated based on the total contaminant mass entering the network and, consequently, the potential exposure to users. Future studies should explore additional risk criteria, such as the likelihood of specific pipes failing, as well as the presence of priority buildings like schools and hospitals, to refine sensor placement strategies and enhance contamination detection in critical areas.

The comparison between Table 2 and Table 3 highlights two key differences that strongly influence detection probabilities. First, Table 2 assumes generic sensors, while Table 3 considers realistic sensors targeting *E. coli* and chlorine, each with defined detection limits. Second, in Table 2, contamination is modeled as a non-reactive tracer, meaning no disinfection is present and the contaminant is assumed to move freely through the system. In contrast, Table 3 incorporates chlorine disinfection, allowing for interactions between chlorine and *E. coli*, which significantly affect contaminant behavior. This comparison underscores that both the sensor model and the contaminant’s chemical reactivity can substantially influence detection performance, demonstrating the limitations of oversimplified modeling assumptions and of relying on generic sensor systems [34].

While this section provides a more detailed analysis of specific sensor performances, certain assumptions remain. All sensors are assumed to operate with a 5 min detection time step, which is a reasonable approximation for pressure and chlorine sensors, but less applicable to *E. coli* detection. For instance, the ColiMinder systems detect microbiological contamination within 15 min by measuring the enzymatic activity and are characterized by a detection limit of 0.8 mMFU/100 mL, which may still pose challenges for identifying low *E. coli* levels in WDNs. While in situ *E. coli* monitoring has shown promise in wastewater and surface water applications [35], its effectiveness for drinking water networks remains an area requiring further development [7,36]. It is important to note that the intrusion scenarios simulated here are based on single-node intrusions resulting from pipe break events. However, the same approach could be extended to more complex combinations of scenarios. Future analyses should consider more complex scenarios involving multiple simultaneous breaks, widespread contamination sources, or additional demand surges that could further exacerbate pressure drops, chlorine demand and increase the risk of contaminant intrusion.

Beyond detection performance, both the choice of sensor placement strategy and the type of sensors deployed carry important economic and practical implications for water utilities. Topological approaches offer advantages in terms of computational simplicity and rapid implementation, making them attractive when technical resources are limited, or fast deployment is needed. In contrast, optimization-based methods typically provide higher detection accuracy, particularly under scenario-specific conditions, but require an updated and calibrated hydraulic model, significantly greater computational time, and may be less robust when system conditions are uncertain. Similarly, the type of sensor influences both performance and feasibility: pressure sensors are more affordable, easier to install, and better suited for large-scale deployment. Chlorine sensors are valuable for ensuring regulatory compliance, maintaining adequate residual levels, and guiding disinfectant dosage adjustments. However, in the simulated intrusion scenarios, the chlorine concentration changes induced by contamination events were minimal, limiting the effectiveness of chlorine sensors for *E. coli* intrusion detection. In chlorinated conditions (AC-C), *E. coli* sensors resulted in low detection probabilities, due to the inactivation by chlorine, whereas in non-chlorinated systems, the efficiency was higher (AC-NC).

## 5. Conclusions

This study evaluates different approaches for designing a monitoring system in WDNs, considering various contamination scenarios: intentional and accidental intrusions modeled as a conservative tracer, and accidental contamination in a chlorinated system, where interactions between disinfectant and *E. coli* is accounted for. A novel methodology for accidental contamination modeling is introduced, simulating low-pressure events caused by pipe breaks that create pressure gradients enabling contaminant ingress. Unlike most previous studies that assume a predefined intrusion flow rate, this approach dynamically estimates it based on the available pressure at the intrusion node. Additionally, this study goes beyond the common assumption of using generic sensors, assessing the performance of specific sensor types and analyzing how sensor threshold settings influence detection probabilities. The key findings of this study are as follows:

- The choice of weighting factors significantly impacts sensor placement performance when 10 or 25 sensors are placed, as reflected by the variability in *DP* across different weights. However, with 100 sensors, the impact of weighting factors diminishes, and network connectivity becomes the dominant factor. Future studies should explore whether this trend holds across different WDN configurations and determine at what sensor density weighting effects become negligible.
- Although optimization-based sensor placement consistently demonstrates superior performance over topological methods, this advantage becomes less pronounced with a higher number of sensors or when the system is evaluated against scenarios that differ from those used during optimization. This suggests that topological methods provide a viable alternative, particularly when there is high uncertainty about contamination types or when a fully calibrated WDN model is unavailable, making optimization impractical.
- The performance of the sensor system varies depending on the contamination scenarios simulated. For water utilities, a practical approach could be to focus on the most vulnerable areas, such as regions with lower pressure, lower residual disinfectant, or higher susceptibility to contamination, and tailor sensor placement to these specific zones. This targeted approach can optimize detection and response.
- In the studied WDN, continuous chlorination effectively limits the impact of *E. coli* intrusion, leading to rapid inactivation without significantly altering chlorine concentrations or causing widespread depletion of chlorine residuals.
- The detection of *E. coli* and chlorine variations in single-node contamination events caused by pipe breaks is highly challenging in chlorinated networks. The rapid inactivation of *E. coli* limits its effectiveness as an indicator of contamination in these systems.
- The rapid inactivation of *E. coli* and the limited drop in chlorine residual during single-node pipe break events reduce the effectiveness of monitoring *E. coli* and chlorine as indicators of contamination in the studied scenarios. In contrast, pressure sensors consistently demonstrated high detection performance across both topological and optimization-based placements, making them a suitable choice for monitoring in such conditions.
- The choice of sensor type and detection thresholds significantly influences detection performance. Defining accurate sensor thresholds is critical not only during sensor development and testing but also in real-time data analysis to minimize false positives and false negatives.
- This study highlights the trade-offs between computational efficiency and detection accuracy in sensor placement strategies and underscores the importance of considering both hydraulic and water quality dynamics when designing monitoring systems for WDN using optimization approaches.

## Figures and Tables

**Table 1:** Overview of the weighting factors, including formulas and key considerations. Parameters: *L* (pipe length), *D* (pipe diameter), $L_{tot}$ (total WDN length), and *Z* (pipe elevation).

Weight	Diameter	Inverse of Diameter	Hydraulic Conductance (HC) ^1^	Relative Length (RL) ^2^	Inverse of RL	Length	Inverse of Length	Elevation ^3^
Formula	D	1/D	$\frac{D^{5}}{L}$	$\frac{L}{L_{tot}}$	$\frac{L_{tot}}{L}$	L	1/L	Z

^1^ The opposite of hydraulic resistance. Resistance coefficient, in commonly used formulas, is a function of ${L\;*\;D}^{\approx -5}$ (Hazen–Williams, Darcy–Weisbach, Chezy–Manning). ^2^ RL provides insight into the probability of a segment experiencing service interruptions, necessitating repair due to breakage [22]. ^3^ The elevation of the pipe is determined by the central point’s elevation.

**Table 2:** *DP* and CV of sensor systems across different approaches for IC and AC-NC.

	Scenarios Set IC			Scenarios Set AC-NC		
**Number of sensors**	#10	#25	#100	#10	#25	#100
**Topological Approaches**						
**No weight**	41.38	70.21	74.71	44.88	64.79	77.12
**Diameter**	37.35	54.87	78.25	44.25	63.27	78.07
**Inverse of Diameter**	44.39	69.31	78.89	48.67	74.97	75.54
**HC**	41.96	56.51	79.52	43.17	66.69	79.01
**RL**	40.63	64.29	80.53	47.28	74.53	76.68
**Inverse of RL**	43.12	65.87	79.15	49.62	54.61	78.57
**Length**	43.70	62.86	73.55	52.53	62.14	71.56
**Inverse of length**	43.86	69.05	79.52	53.79	58.79	79.71
**Elevation**	37.41	69.89	79.79	44.31	64.16	78.82
**CV [%]**	6	9	3	8	10	3
**Optimization Approaches**						
**Optimization based on scenarios set IC**	77.25	80.26	85.45	73.07	77.88	86.98
**Optimization based on scenarios set AC-NC**	76.03	72.27	83.23	78.63	80.34	88.62

**Table 3:** DP with 10 sensors across sensor types and thresholds for topological (RL, 1/RL) and optimization approaches.

Parameter Monitored	Pressure	*E. coli*		ΔCl_2_	
**Detection threshold**	3 m	0 CFU/L	0.1 CFU/L	0.2 mg/L	0.3 mg/L
**Topological Approaches**					
**RL**	93	3	0	3	2
**Inverse of RL**	91	4	0	3	2
**Optimization Approaches**					
**Optimized for pressure-based DP**	100	23	6	3	0
**Optimized for chlorine-based DP ** **(0.2 mg/L)**	63	14	4	11	2
**Optimized for chlorine-based DP ** **(0.3 mg/L)**	91	5	3	4	4
**Optimized for *E. coli*-based DP ** **(0.1 CFU/L)**	93	57	38	3	1

## Data Availability

Data available on request due to restrictions.

## Supplementary Materials

The following supporting information can be downloaded at https://www.mdpi.com/article/10.3390/s25175320/s1, Figure S1: Conceptual framework illustrating the modeling procedure from pipe break simulation to single-point intrusion modeling using fictitious reservoirs. Figure S2: Graphical representation of DP across sensor placement methods. Figure S3: Bar charts of detection probability by sensor type, threshold, and method (SCENARIO AC-C). Table S1: DP with 10 sensors across sensor types and thresholds for topological and optimization approaches.

## Abbreviations

The following abbreviations are used in this manuscript:

WDN	Water Distribution Network
HC	Hydraulic Conductance
RL	Relative Length
GA	Genetic Algorithm
DP	Detection Probability
FCV	Flow Control Valve
CV	Coefficient of Variation
