# Supplementary material for: Design of Monitoring Systems for Contaminant Detection in Water Networks Under Pipe Break-Induced Events
Source: Sensors (Basel). 2025 Aug 27;25(17):5320. doi: 10.3390/s25175320 (PMC12431176; doi:10.3390/s25175320)
Supplement: Supplementary file 1 [file sensors-25-05320-s001.zip › sensors-3788140-supplementary.pdf]

SUPPLEMENTARY MATERIAL

Design of Monitoring Systems for Contaminant Detection in Water Networks Under Pipe Breaks Induced Events

Ludovica Palma[1]*, Fatemeh Hatam[2], Armando Di Nardo[3] and Michèle Prévost[4]

[1] Industrial Chair on Drinking Water, Department of Civil, Geological and Mining Engineering, Polytechnique Montréal, CP 6079, Succ. Centre-ville, Montréal, QC H3C 3A7, Canada; ludovica.palma@polymtl.ca
[2] Industrial Chair on Drinking Water, Department of Civil, Geological and Mining Engineering, Polytechnique Montréal, CP 6079, Succ. Centre-ville, Montréal, QC H3C 3A7, Canada; fatemeh-2.hatam@polymtl.ca
[3] Department of Engineering, Università della Campania Luigi Vanvitelli, Aversa 81031, Italy; armando.dinardo@unicampania.it
[4] Industrial Chair on Drinking Water, Department of Civil, Geological and Mining Engineering, Polytechnique Montréal, CP 6079, Succ. Centre-ville, Montréal, QC H3C 3A7, Canada; michele.prevost@polymtl.ca
* Correspondence: ludovica.palma@polymtl.ca

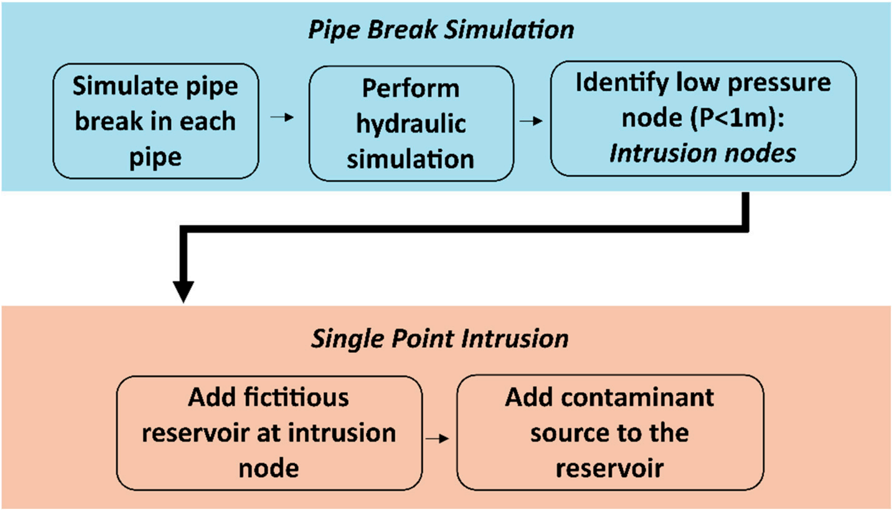

Figure S1. Conceptual framework illustrating the modeling procedure from pipe break simulation to single-point intrusion modeling using fictitious reservoirs.

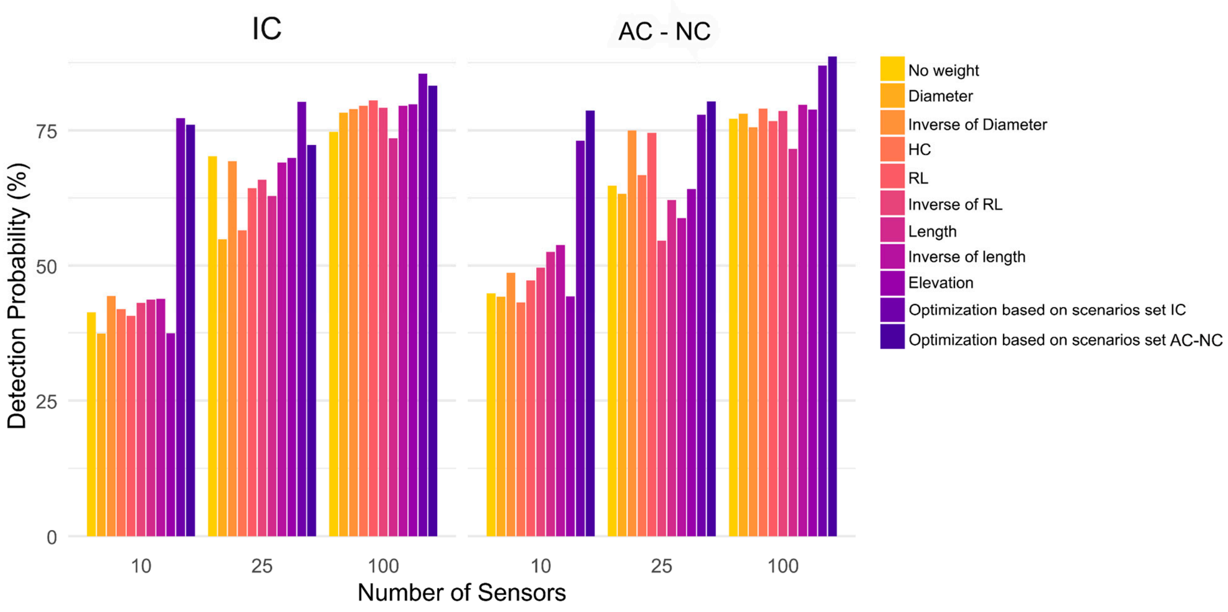

Figure S2. Graphical representation of *DP* across sensor placement methods.

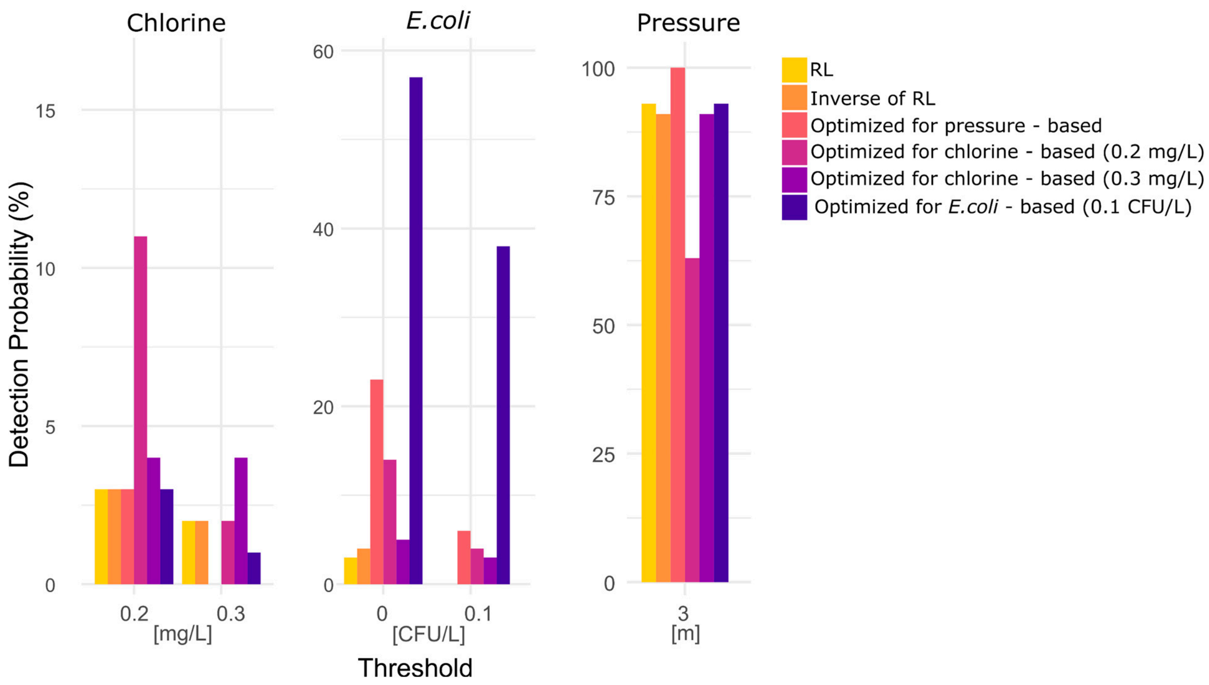

Figure S3. Bar charts of detection probability by sensor type, threshold, and method (SCENARIO AC-C)

Table S1. *DP* with 10 sensors across sensor types and thresholds for topological and optimization approaches.

Parameter Monitored	Pressure	*E. coli*		ΔCl_2	
**Detection threshold**	3 m	0 CFU/L	0.1 CFU/L	0.2 mg/L	0.3 mg/L
Topological Approaches					
**No weight**	91	3	0	3	2
**Diameter**	80	3	0	3	2
**Inverse of Diameter**	91	3	0	3	0
**HC**	91	3	0	3	0
**RL**	93	3	0	3	2
**Inverse of RL**	91	4	0	3	2
**Length**	93	3	0	3	2
**Inverse of length**	91	4	0	3	2
**Elevation**	90	3	0	3	2
**Standard Deviation [%]**	3.9	0.4	0	0.3	0.9
Optimization Approaches					
**Optimized for pressure-based *DP***	100	23	6	3	0
**Optimized for chlorine-based *DP* (0.2 mg/L)**	63	14	4	11	2
**Optimized for chlorine-based *DP* (0.3 mg/L)**	91	5	3	4	4
**Optimized for *E. coli*-based *DP* (0.1 CFU/L)**	93	57	38	3	1
